# PEOT/PBT Polymeric Pastes to Fabricate Additive Manufactured Scaffolds for Tissue Engineering

**DOI:** 10.3389/fbioe.2021.704185

**Published:** 2021-09-14

**Authors:** Gustavo A. Higuera, Tiago Ramos, Antonio Gloria, Luigi Ambrosio, Andrea Di Luca, Nicholas Pechkov, Joost R. de Wijn, Clemens A. van Blitterswijk, Lorenzo Moroni

**Affiliations:** ^1^ Institute for BioMedical Technology and Technical Medicine (MIRA), Tissue Regeneration Department, University of Twente, Enschede, Netherlands; ^2^ Institute of Ophthalmology, University College of London, London, United Kingdom; ^3^ Institute of Polymers, Composites and Biomaterials, National Research Council of Italy, Naples, Italy; ^4^ MERLN Institute for Technology-inspired Regenerative Medicine, Complex Tissue Regeneration Department, Maastricht University, Maastricht, Netherlands

**Keywords:** microparticles, additive manufacturing, tissue engineering, polymers, mesenchymal stem cells, mechanical analysis

## Abstract

The advantages of additive manufactured scaffolds, as custom-shaped structures with a completely interconnected and accessible pore network from the micro- to the macroscale, are nowadays well established in tissue engineering. Pore volume and architecture can be designed in a controlled fashion, resulting in a modulation of scaffold’s mechanical properties and in an optimal nutrient perfusion determinant for cell survival. However, the success of an engineered tissue architecture is often linked to its surface properties as well. The aim of this study was to create a family of polymeric pastes comprised of poly(ethylene oxide therephthalate)/poly(butylene terephthalate) (PEOT/PBT) microspheres and of a second biocompatible polymeric phase acting as a binder. By combining microspheres with additive manufacturing technologies, we produced 3D scaffolds possessing a tailorable surface roughness, which resulted in improved cell adhesion and increased metabolic activity. Furthermore, these scaffolds may offer the potential to act as drug delivery systems to steer tissue regeneration.

## Introduction

Additive manufacturing systems appear to be one of the most promising techniques to meet many of the general requirements for scaffold fabrication, as they can process a wide range of biomaterials in a custom-made shape with tunable properties (Hutmacher, 2001; Yang et al., 2002; Yeong et al., 2004). Within the additive manufacturing systems, 3D fiber deposition (3DF) has been widely used by our groups to fabricate custom-made scaffolds for tissue engineering applications with encouraging results (Woodfield et al., 2004; Moroni et al., 2006; Woodfield et al., 2006). 3DF is essentially a fused deposition modeling (FDM) technique used for the extrusion of highly viscous molten thermoplastic polymers and biomaterials pastes from a controlled robotic unit to a stage in the form of a fiber, on a layer-by-layer fashion, offering appealing solutions for scaffold fabrication. The standard outcomes are 3D scaffolds with fine tailorable porosity, pore size and shape, with completely interconnected pore network that allows for better cell migration and nutrient perfusion to the deeper parts of the constructs when compared to the 3D architectures fabricated using conventional techniques (Sachlos et al., 2003; Malda et al., 2004). In contrast, conventional techniques require a percolating distribution of a porogen agent to achieve a porous network (Moroni et al., 2005), which is often tortuous and not completely interconnected.

Microsphere-based scaffold fabrication approaches have attracted great deals of attention as they provide outstanding mechanical properties and allow for the controlled release of bioactive molecules (Shi et al., 2011). They have been widely used for drug delivery applications mainly due to their ability to enhance the release efficacy of the encapsulated drug as they provide larger surface area to volume ratios as well as spatial and temporal control over drug release (White et al., 2013; Rahman et al., 2014; Gupta et al., 2017). In addition, they are rigid structures that can be closely packed together, alone or in combination with other materials to yield porous 3D structures to act as tissue engineering scaffolds. The densely packed microsphere-based scaffolds can act both as a guide for establishing intricate cell–cell and/or cell–ECM interactions (Zhu et al., 2007) or serve as a template to induce cell proliferation (Belkas et al., 2004; Valmikinathan et al., 2008). By incorporating microspheres on the 3D constructs, one can generate a network of pores inside the interior of a scaffold, which facilitates cellular ingrowth and accelerates scaffold resorption (Dellinger et al., 2006; Habraken et al., 2006; De Nardo et al., 2012). Microsphere based scaffolds can be categorized as either 1) injectable or 2) sintered scaffolds. The latter shows greater advantages as the microspheres are fused together to create a single macroscopic unit, which prevents the microspheres from flowing out from the defect size upon implantation. In contrast, injectable microsphere-based scaffolds exist as a liquid suspension that acquires the shape of the defect following implantation. Sintered microsphere-based scaffolds have been used to engineer scaffolds for different applications, including bone (Borden et al., 2002) and cartilage regeneration (Singh et al., 2008; Dormer et al., 2010; Dormer et al., 2011; Dormer et al., 2012; Dormer et al., 2013). An extensive review on the main advantages and disadvantages of each microsphere-based scaffold type, their fabrication methods, design strategies, and applications can be found at Gupta et al. (2017). Cell attachment, morphology, proliferation, and differentiation are all processes affected by the physical (stiffness, roughness, fiber diameter, topography) and chemical (surface energy, wettability) structures of the surrounding material and have been extensively addressed and reviewed elsewhere (Mahmood et al., 2004; Jansen et al., 2005; Dhirendra et al., 2008; Ahmed et al., 2015; Tallawi et al., 2015; Ahmed et al., 2018). Hence, the aim of this study was to fabricate distinct types of 3D microspherical pastes with tailorable fiber diameter and surface topography. For this purpose, we used 3DF to fabricate microsphere-based scaffolds mixed with different polymeric biocompatible binders sintered at different temperatures. Microspheres were composed of block-copolymers of poly(ethylene oxide terephthalate) (PEOT) and poly(butylene terephthalate) (PBT). These polyether-ester multiblock copolymers belong to a family of thermoplastic elastomers that has been extensively used for tissue engineering and drug delivery applications (Deschamps et al., 2002; van Dijkhuizen-Radersma et al., 2002; Olde Riekerink et al., 2003; Mahmood et al., 2004) and used in clinical applications (PloyActive™, IsoTis Orthopaedics S.A.) as a cement stopper and bone filler for orthopedic surgeries (Bulstra et al., 1996; Mensik et al., 2002). Poly(caprolactone) (PCL), here used as a polymeric binder, has been considered as one of the standard polymers for tissue engineering applications due to its good biocompatibility, bioresorbability, and mechanical properties (Wang, 1989; Peluso et al., 1997). Additionally, biphasic calcium phosphate (BCP) particles were added to the paste formulation given their optimal properties to be used in tissue engineering approaches, particularly for osteoconductive and osteoinductive applications (Rice et al., 2003; Habibovic et al., 2006a; Habibovic et al., 2006b). These properties are controlled by the ratio of their two constitutive phases (a more stable hydroxyapatite (HA) Ca_10_(PO_4_)_6_(OH)_2_ phase and a more soluble beta-tricalcium phosphate (β-TCP) Ca_3_(PO_4_)_2_ phase). Alginate was also incorporated in this study, as a second phase polymer binder, to attempt to construct cell-laden networks that can easily be incorporated in the site of the defect. Alginate has excellent biocompatibility, biodegradability, non-antigenicity and chelating properties and has been widely used in the preparation of cell free and cell laden hydrogels, but also in the form of microspheres for tissue repair and regeneration applications. An extensive review on the properties and biomedical applications of alginate can be found at Lee and Mooney (2012) and Sun and Tan (2013).

The hybrid structures here proposed hypothetically combine the flexibility of polymers with the mechanical strength of ceramics, while maintaining the individual physico-chemical and biological properties of the PEOT/PBT copolymers, PCL, alginate and BCP particles. Together with the aforementioned advantages of microspheres-based scaffolds, these are great assets to architecture tissue-engineered based scaffolds. Sintering temperature and time, the two major factors that affect the mechanical properties and porosities of the sintered microsphere-based scaffolds, were also tuned in order to fabricate scaffolds with different topographies and variable degree of microsphere blending. Mesenchymal stromal cells were obtained from human bone marrow biopsies and used as models to assess the effect of scaffold structure on cell metabolic activity and morphology.

## Materials and Methods

### Scaffolds Fabrication

Poly(ethylene oxide—terephthalate)/poly(butylene terephthalate) (PEOT/PBT) copolymers were kindly provided by PolyVation (Groningen, Netherlands). The composition used in this study was 300PEOT55PBT45 where, following an aPEOTbPBTc nomenclature, “a” is the molecular weight in g/mol of the starting PEG blocks used in the copolymerization, while “b” and “c” are the weight ratios of the PEOT and PBT blocks, respectively. Microspheres of 300PEOT55PBT45 (300/55/45) were fabricated using a water-in-oil-in-water emulsion method, as previously described (Sohier et al., 2003). Briefly, a 12.5% wt/v 300/55/45 solution in chloroform (Sigma) was emulsified in water using an Ultra-Turrax (IKA-Labortechniek) for 30 s at 19,000 rpm. The emulsion was poured in a 4% wt/v poly(vinyl alcohol) solution in PBS and stirred at 400 rpm for 5 min. The solution was then added with 1 L of demineralized water and stirred overnight to let the solvent evaporate. Microspheres were collected by centrifugation, washed with PBS three times, sieved into two different size populations, namely below 64 µm and between 64 and 128 μm, and freeze-dried.

**FIGURE 1:**
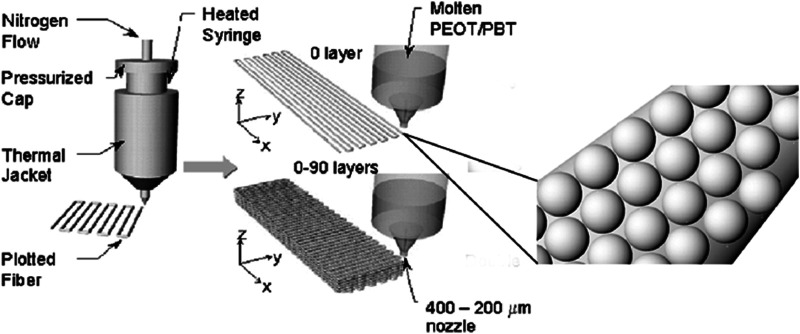
Schematic showing the 3D fiber deposition process. A colloidal solution of microspheres embedded into a second polymeric carrier X is extruded into a computer-aided design pattern through a nozzle of 200–400 µm internal diameter by applying a nitrogen pressure. The syringe containing the colloidal solution can be heated or maintained at room temperature. Adapted from Woodfield et al. (2004).

In general, polymeric pastes of 300/55/45 microspheres and a second polymeric phase X were made by vigorously mixing the microspheres in a 10% wt/v solution of the polymer X, acting as a binder. The microsphere:binder mixing ratio was 1.75:1. Poly(vinyl pyrrolidone) (PVP) (Sigma-Aldrich), poly(caprolactone) (PCL) (Sigma-Aldrich), and alginate (Sigma-Aldrich) were used as a second polymeric phase “X”. PVP and alginate were dissolved in deionized water, while PCL in acetic acid (Sigma-Aldrich). In addition, a further strategy was considered showing the possibility to add bioactive components in the pastes. In particular, 300/55/45 microspheres were first added to a PCL-acetic acid solution (10% wt/v) and vigorously mixed. Successively, BCP nanoparticles (10% by weight with respect to the amount of polymeric microspheres) were also added to 300/55/45-PCL paste formulations. 3DF scaffolds were fabricated by using a Bioplotter (Envisiontec, Germany). In brief, the pastes were loaded into a syringe at room temperature and extruded through a nozzle at a pressure of 1–2 bar, depending on the paste viscosity. The pastes were plotted in a computer-controlled pattern on a stage as layers of fibers (Figure 1). A tapered nozzle of internal diameter of 0.4 mm was used. Fiber spacing was maintained at 1,000 μm, while layer thickness at 300 µm. Scaffolds with a smooth surface were manufactured by conventional processing of 300/55/45, using the method previously described by Moroni et al. (2006). Briefly, polymer granules were inserted in the cartridge of the Bioplotter, heated at 190°C and extruded through the nozzle after applying a pressure of 5 bar. The nozzle had an internal diameter of 0.4 mm. Fiber spacing was maintained at 1,000 μm, while layer thickness at 250 µm.

Scaffold architecture included deposition of fibers in each layer at 90° angles of the successive layers for cell culture studies.

### Scanning Electron Microscopy Analysis

The final structures were characterized by scanning electron microscopy (SEM, Philips XL 30 ESEM-FEG). Acellular scaffolds were dried, and gold sputtered (Cressington) prior to analysis. The scaffolds from the *in vitro* studies were dehydrated in sequential ethanol series (50 vol%, 60 vol%, 70 vol%, 80 vol%, 90 vol%, 96 vol%, 100 vol% in ultrapure water (MiliQ water), and dried with a critical point drier (Balzer).

### Mechanical Compression Tests

Compression tests were performed on 3DF scaffolds based on PEOT/PBT polymeric pastes with or without BCP nanoparticles using block-shaped specimens (6 mm × 6 mm × 9 mm). The scaffolds were immersed at 37°C in physiological solution and tested in the wet state at a rate of 1 mm/min up to a strain of 50%, using an INSTRON 5566 testing machine. The stress (*σ*) was evaluated as the measured force F divided by the initial cross-sectional area (A_0_) of the scaffold:

$$\sigma =\frac{F}{A_{0}}$$ (1)

The strain (ε) was calculated as the ratio between the height variation (Δh) of the scaffold and its initial height (h_0_):

$$\varepsilon =\frac{\text{Δh}}{h_{0}}$$ (2)

### Cell Culture

Human mesenchymal stromal cells (hMSCs) were isolated, cultured, and cryopreserved as described by Both et al. (2007). Stromal cells were obtained from donors who were undergoing total hip replacement surgery with previous written informed consent for bone marrow biopsy, all approved by the local medical ethical committee. Mono-nucleated cells were counted in the aspirate and plated at a density of 500,000 cells/cm^2^ in T-flasks (Nunc, Thermo Fischer Scientific, Roskilde, Denmark). After addition of α-minimal essential medium (αMEM) proliferation medium, cells were cultured for four to 5 days. The αMEM proliferation medium contained minimal essential medium (GIBCO, Carlsbad, CA), 10 vol% fetal bovine serum of a selected batch (FBS; Biowhittaker, lot:8SB0002; from Lonza, Verviers, Belgium), 0.2 mM L-ascorbic-acid-2-phosphate (Sigma, St. Louis, MO), penicillin G (100 Units/ml, Invitrogen, Carlsbad, CA); streptomycin (100 μg/ml, Invitrogen), 2 mM L-glutamine (Sigma-Aldrich), and 1 ng/ml basic fibroblast growth factor (Instruchemie, Delfzijl, Netherlands). Cells were cultured in a tissue incubator at 37°C in a humidified atmosphere of 5% carbon dioxide. After the four to 5 days culture period, non-adherent cells and αMEM proliferation media were discarded. Adherent cells were thoroughly washed twice with phosphate-buffered-saline (PBS, Sigma-Aldrich) and αMEM proliferation medium was refreshed. Adherent cells were proliferated for two passages and thereafter cryopreserved. The passage number was defined by every harvest with 0.25% trypsin/EDTA (GIBCO).

Cryopreserved cells were thawed—passage 2—recounted and plated at a density of 1,000 cells/cm^2^ in 300 cm^2^ T-flasks (T-300 flasks) in αMEM proliferation media. Stromal cells were cultured for 1 week with one refreshment of αMEM proliferation media.

3D scaffolds were sterilized with 70 vol% ethanol in ultrapure water solution for 15 min. Thereafter, scaffolds were washed and incubated at room temperature for 2 h with sterile PBS. This washing step was repeated three times. Scaffolds were then incubated in αMEM proliferation media overnight at the standard culture conditions before seeding.

Stromal cells were harvested with 0.25 vol% trypsin/EDTA after reaching sub-confluency. From the cell suspension, 200 µL were diluted in 10 ml of Isoton II diluent (Beckman Coulter, Fullerton, CA) and three drops of Zap-O Globin II lytic reagent (Beckman Coulter) were added. The solutions were incubated for 30 min to maximize the effect of the lytic reagent, and subsequently, cell nuclei were counted in a particle count and size analyzer (Z2, Beckman Coulter). The size range of counted nuclei was set between 6 and 10.5 µm according to the 95% confidence interval of stromal cells nuclei size. After cell counting, 2 × 10^5^ stromal cells—passage 3—were seeded per scaffold and adjusted to 1 ml/well in non-tissue-culture treated plates (10 cm^2^ per well, Nunc). To induce homogeneous seeding, the six-well plates were placed on a rocking bed (Heidolph Instruments, Schwabach, Germany) at 30 rpm for 4 h in standard culture conditions. The plates were thereafter removed from the rocking bed and placed in an incubator. Medium was refreshed every other day, and a medium sample was withdrawn for metabolic profiling.

### Cell Viability

Cell viability was assessed using alamarBlue™ Reagent according to the manufacturer’s protocol (ThermoFisher Scientific). Briefly, 10 vol% of alamarBlue™ reagent was added in each well (*n* = 3) and incubated at 37°C for 2 h. Three 100 µl media samples were transferred from each well into a Nunc™ 96-well plate (ThermoFisher Scientific). Fluorescence was measured at 540–570 nm excitation 580–610 nm emission in VICTOR3™ 1,420 Multilabel Counter (PerkinElmer). The readout from the scaffolds was corrected with a blank (fresh medium plus alamarBlue™ reagent).

### Metabolic Activity

Media samples were obtained every other day from independent wells (*n* = 3). Glucose and lactate were measured in a Vitros DT60 II chemistry system (Ortho-Clinical Diagnostics, Tilburg, Netherlands), as previously described (Higuera et al., 2015).

### Cell Adhesion and Morphology Assessment

Scaffolds geometry and architecture was characterized by environmental scanning electron microscopy (ESEM) analysis with a Philips XL 30 ESEM-FEG. Samples were fixed overnight in 0.14 M cacodylate buffer (pH = 7.2–7.4) containing 1.5% glutaraldehyde (Merck). Scaffolds were subsequently dehydrated in sequential ethanol series, and critical point dried from liquid carbon dioxide using a Balzers CPD 030 machine. Samples were then gold sputtered and studied under the SEM.

Confocal laser scanning microscopy (CLSM) was carried out to analyze cell adhesion and spreading at 1, 3, and 7 days after seeding, using a Zeiss LSM 510/ConfoCor 2 system (Oberkochen, Germany) equipped with argon and helium–neon lasers. Briefly, cell-seeded scaffolds were fixed with 4% paraformaldehyde in PBS at room temperature for 30 min and washed with PBS. Then, samples were permeabilized with 0.25% Triton X-100 in PBS for 1 h and blocked with 1% bovine serum albumin (BSA) for 30 min. F-actin was stained with rhodamine phalloidin (1:40 v/v in 1% BSA/PBS) (Invitrogen) for 1 h. The 543 nm helium-neon laser was used for rhodamine excitation. Thus, the actin filaments were visualized. The CLSM images were successively analyzed with ImageJ software (NIH, Bethesda, MD, United States) to further evaluate the cell morphology. The cell shape factor (*Ф* = 4πA/P^2^) was also determined based on the area (A) and the perimeter (P) of a cell.

### Alkaline Phosphatase Activity and Glycosaminoglycans Quantification

Samples were removed from the medium and washed twice with PBS at 7, 14 and 21 days. The cell-scaffold constructs were then incubated in lysis buffer and centrifuged. To evaluate the differentiation towards early osteogenic lineage, the alkaline phosphatase (ALP) activity was measured using the SensoLyte pNPP alkaline phosphatase assay kit (AnaSpec Inc., Fremont, CA, United States). To evaluate the differentiation toward early chondrogenic lineage, the GAG amount was quantified using 1,9-Dimethyl Methylene Blue (DMMB) assay. 25 μl of sample were placed into a transparent flat bottom 96 well plate and 5 μl of 2.3 M NaCl solution were added, then 150 μl of DMMB solution were added and absorbance was read using a Multiscan Go (Fisher Scientific, Landsmeer, the Netherlands) plate reader at a wavelength of 525 nm. GAG content was quantified with a chondroitin standard curve. DNA was also detected and quantified by the Quant-iT PicoGreen assay kit (Molecular Probes Inc., Eugene, OR, United States). Thus, the normalized ALP activity (ALP/DNA) and GAG content (GAG/DNA) were determined. All the solutions were prepared following the manufacturer’s protocol and a well-defined procedure. The experiments were performed at least three times in triplicate.

### Statistical Analysis

For each experiment, three biological replicates of each condition were used and statistical analysis was performed using SPSS Statistics 18.0, with One Way ANOVA and Tukey’s Multiple Comparison test or Student *t*-Test. Data are presented as mean ± standard deviation and statistical significance was determined for *p* < 0.05.

## Results and Discussion

### Scaffolds Characterization

**FIGURE 2:**
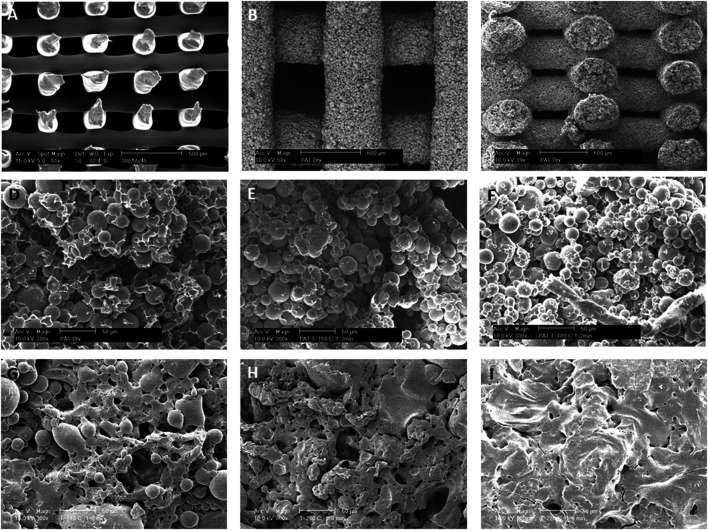
3D deposited scaffolds fabricated by **(A)** melting extrusion of 300PEOT55PBT45 thermoplastic polymers or **(B–F)** extrusion of 300PEOT55PBT45-PVP microspheres polymeric pastes at room temperature. **(B,C)** Top and cross section views of fabricated scaffolds from polymeric pastes showing a completely interconnected pore network. **(D–F)** Microspheres are loosely bound after **(D)** drying at room temperature or coalesced after “soft” sintering at **(E)** 150°C and **(F)** 180°C for 1 minute. **(G–I)** The effects of sintering temperatures on microsphere coalescence. 300PEOT55PBT45-PVP pastes were sintered for 8 min at **(G)** 170°C, **(H)** 200°C, and **(I)** 220°C. Scale Bar: **(A–C)** 500 μm; **(D–I)** 50 µm.

Two pastes were prepared: either composed of microspheres with dimensions below 64 µm or composed of microspheres with dimensions between 64 and 128 µm (Supplementary Figure S1). Due to the larger size of microspheres in the latter that led to nozzle clogging, scaffolds were often depicted with defected microspheres (Supplementary Figure S1B), and therefore excluded from this study. Microspheres in the two fabricated pastes (64 μm and 64–128 µm) had an average diameter of 26 ± 9 μm and 47 ± 31 μm, respectively. Scaffold shrinkage after drying appeared below 12% across all conditions and dependent on temperature (Supplementary Figure S2A) and sintering time (time during which the microspheres were left to bind to each other at a given temperature (Supplementary Figures S2B,C). The low levels of shrinkage, particularly at low sintering times and temperature for the smaller microspheres, suggest little deformation upon treatment, and therefore proportionally big pore sizes and high porosities. Figure 2 shows a comparison between smooth 3DF scaffolds fabricated with molten 300/55/45 (Figure 2A) and with 300/55/45-PVP based pastes (Figures 2B–F). Below the melting temperature (T < 180°C) of the 300/55/45-PVP based pastes, microspheres only partially melted to each other (Figures 2D–F). On the other hand, above its melting temperature (T > 180°C), the microspheres melted into each other, resulting in an augmented coalescence with the increasingly sintering time from 1 to 8 min. This also resulted in different surface morphologies; from well distinguishable individual spheres at lower sintering temperatures (Figure 2G) to completely melted surfaces at higher sintering temperatures (Figure 2I). Sintering time and temperatures are the two main factors that affect the mechanical properties and porosities of the sintered microsphere-based scaffolds. High temperatures and long sintering times have equivalent effects on the properties of scaffolds; with greater fusion between the spheres that leads to a decrease in pore size and volume and an increase in scaffold’s compressive modulus. The greater degree of microsphere fusion results in possible closure of the pores, reducing the interconnectivity and therefore the overall volume of the scaffold (Jiang et al., 2006; Shi et al., 2011). This leads to a reduction in nutrient diffusion to the inner scaffold and eventually a lost in function. Our results showed that moderate sintering temperatures of 150 and 180°C (below the 300/55/45-PVP pastes melting point) and sintering time of 1 minute led to moderate degrees of sphere binding with no pore closure nor loss of interconnectivity. Additionally, when the sintering times were increased to 8 min with increasing temperatures (above the 300/55/45-PVP pastes melting point), a remarkable sphere melting, and loss of interconnectivity could be appreciated. Taken together these results suggest that temperatures (below the constituent polymer’s melting point) and intermediate sintering times should be employed in the fabrication of these structures.

**FIGURE 3:**
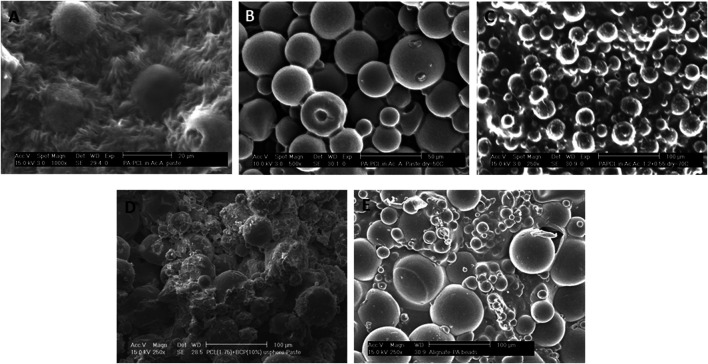
Different surface morphologies were obtained when PCL solutions were used as the second polymeric phase and dried at different temperatures: **(A)** room temperature, **(B)** just below PCL melting temperature at 50°C, or **(C)** just above PCL melting temperature at 70°C. **(A,C)** Microspheres are entrapped within the PCL matrix or **(B)** loosely bound to each other via a PCL thin neck layer. **(D)** BCP-containing 300PEOT55PBT45-PCL pastes (70°C). **(E)** Alternatively, PCL was substituted as the second polymeric phase, and 300PEOT55PBT45-alginate pastes were created. Scale bars: **(A)** 20 μm; **(B)** 50 μm; **(C–E)** 100 µm.

We then investigated the effect of a different second polymeric phase (PCL) on the scaffold surface topography. When PVP was replaced by PCL, further modifications on the 300/55/45 microspheres binding were achieved at lower sintering temperatures. Namely, when acetic acid was left to evaporate at room temperature, the 300/55/45 microspheres were embedded within the PCL matrix with distinct surface roughness, and no clear individual spheres were appreciated (Figure 3A). When the drying process was facilitated by increasing the environmental temperature to 50°C or 70°C, which are right below or above the melting temperature of PCL, microspheres were just bound neck-to-neck by a thin layer of PCL (Figure 3B) or embedded in a smooth thin PCL matrix (Figure 3C) with clear individual microspheres observed. These observations further corroborate the effect of sintering temperatures on scaffold’s topography. Additionally, these results showed that different polymers can be used to lower the sintering temperatures to allow 1) the interconnectivity of the scaffold and 2) the incorporation of bioactive molecules within the microspheres as high temperatures may compromise the bioactivity of such components. To do so, BCP was added to the 300/55/45-PCL pastes to create bioactive microsphere scaffolds with a certain degree of coalescence and rougher surfaces (Figure 3D) when compared to those where BCP was not utilized. BCP has been broadly used in tissue engineering, mainly for musculoskeletal applications, to modulate stem cell differentiation (Lobo et al., 2015; Saldaña et al., 2009; Davison et al., 2015), cell proliferation (Davison et al., 2015; Nath et al., 2013), and cell viability (Nath et al., 2013). In addition to its self-potential to affect cell behavior, the rougher surface of the BCP-containing scaffolds may also be an important physical cue to enhance cell adhesion and proliferation, as previously suggested by Ibrahim et al. (2016). As a last approach, 300/55/45 pastes were created by replacing PCL with alginate to create a hydrogel model (Figure 3E). This results in coalescent individual microspheres covered in a smooth thin alginate matrix. The main advantage of this approach is the possibility of using these pastes as cell-laden networks that can easily be incorporated in the site of the defect. The laden cells could be patient specific (using for example induced pluripotent stem cells), which brings huge advantages to this approach (Takahashi and Yamanaka, 2013; Singh et al., 2015a), and the moderate degree of coalescence makes this structure a macroscopic unit preventing the microspheres from flowing out upon implantation.

We further characterized the mechanical behavior of 300/55/45-PCL pastes with or without BCP nanoparticles. A mechanical behavior similar to flexible foams was obtained from compression tests (Supplementary Figure S3), where stress-strain curves displayed an initial linear region evidencing a stiffer mechanical response at the onset, which was followed by a region with lower stiffness. The compressive modulus and maximum stress were measured, and typical values for 300/55/45-PCL and BCP-containing 300/55/45-PCL scaffolds are reported in Table 1.

**TABLE 1:** Compressive mechanical properties of 3DF scaffolds based on PEOT/PBT polymeric pastes: compressive modulus (E) and maximum stress (σ_max_) reported as mean value ± standard deviation for structures fabricated with 300PEOT55PBT45-PCL and BCP-containing 300PEOT55PBT45-PCL pastes.

Scaffold	E (MPa)	σ_max_ (MPa)
300PEOT55PBT45-PCL	20.1 ± 2.5	4.0 ± 0.3
BCP-containing 300PEOT55PBT45-PCL	50.2 ± 6.1	8.7 ± 0.6

BCP-containing 300/55/45-PCL scaffolds showed values of compressive modulus (50.2 ± 6.1 MPa) and maximum stress (8.7 ± 0.6 MPa) which were significantly higher (*p* < 0.05) than those achieved for 300/55/45-PCL scaffolds (20.1 ± 2.5 MPa and 4.0 ± 0.3 MPa). Thus, the addition of BCP led to a significant increase of the compressive modulus and maximum stress.

### Cell Activity

**FIGURE 4:**
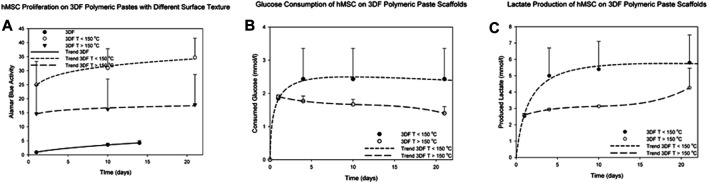
Metabolic activity of hMSCs cultured for up to 21 days on 300/55/45-PVP paste scaffolds. **(A)** The metabolic activity was significantly higher on the paste scaffolds than on smooth scaffolds (thermoplastic polymers). **(B)** Glucose consumption and **(C)** lactate production were measured on 300/55/45-PVP paste scaffolds, indicating a higher glucose consumption and lactate production levels on scaffolds sintered at temperatures lower than 150°C.

Cell metabolic activity was measured over 21 days on 3005545-PVP pastes (composed of microspheres with dimensions below 64 µm) sintered at different temperatures and compared against readings from cells cultured on 300/55/45 smooth thermoplastic polymers. Overall none of the surface modifications showed an adverse effect on hMSCs viability on all the materials tested. Significant increased metabolic activity levels were appreciated as early as 1 day of culture on microsphere scaffolds sintered at a temperature lower than 150°C (*p* < 0.05, Figure 4A) when compared to the other two conditions. These structures corresponded to scaffolds of rougher surfaces and the significant increase can be attributed to a higher surface area of these architectures that introduces higher number of sites for cell adhesion when compared to the smoother fibers. Baki et al. have shown similar results by culturing hMSCs on oxygen plasma modified poly (dl-lactic-co-glycolic acid) (P_DL_LGA) microspheres (Baki et al., 2017). In addition to the higher surface area, these structures have roughened surfaces which has been linked to a melioration on cell adhesion (Ibrahim et al., 2016). Furthermore, the slope of the curves suggests higher proliferation rates of cells cultured in scaffolds sintered at a temperature lower than 150°C when compared to those on scaffolds sintered at a higher temperature or on smooth scaffolds (*p* < 0.05). The latter were kept in culture only for 14 days as the observed cell proliferation was minimal. Further analysis on cell metabolic activity revealed higher glucose consumption and lactate production on scaffolds sintered at temperatures lower than 150°C when compared to those sintered at higher temperatures (Figures 4B,C). In addition to the effect on cell proliferation, cell metabolic activities are also profoundly influenced by the physical cues of the surrounding scaffold (Chang et al., 2003; Singh et al., 2015b; Moerke et al., 2016). The effects can be either beneficial or adverse depending on the cell type and topography pattern. On one hand, Chang et al. have shown that neutrophils cultured on roughened surfaces have decreased cell viability with increased reactive oxygen species production (Chang et al., 2003). On the other hand, Sing et al. showed that astrocytes cultured on micropatterned grooved surfaces have increased mitochondrial activity accompanied by increased ATP release when compared to smooth surfaces (Singh et al., 2015b). Our results suggest that the roughness of these microsphere-based additive manufactured scaffolds promotes an increase in metabolic activity of hMSCs.

**FIGURE 5:**
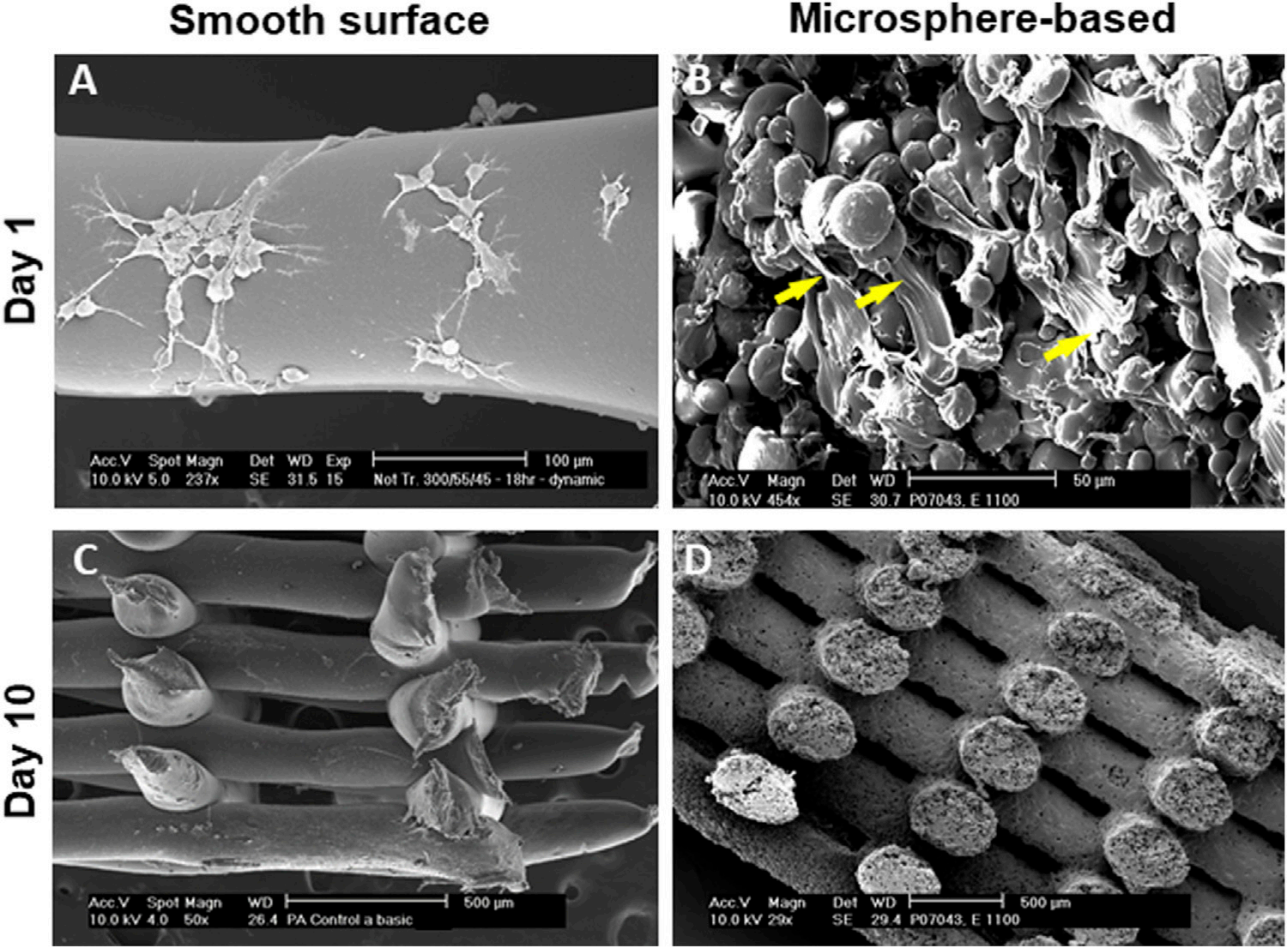
SEM images of cells cultured on 3D fibers. **(A,C)** Cells cultured on smooth scaffolds showed scarce proliferation overtime with round morphologies. **(B,D)** Cells cultured on microspheres-based scaffolds showed higher levels of proliferation with elongated morphologies with numerous filamentous extensions (yellow arrows). Scale bars: **(A)** 100 μm; **(B)** 50 μm; **(C)**; **(D)** 500 µm.

SEM analysis supported these results and revealed that hMSCs, homogeneously covered all the surface of the scaffolds and have an elongated morphology with numerous filamentous extensions (yellow arrows) when cultured on microsphere-based scaffolds. These results suggested that cells proliferate more and better attached to these surfaces. Contrarily, only few cells and with flattened morphology were observed when cultured on smooth scaffolds (Figure 5).

The CLSM analysis on the cell-scaffold constructs further confirmed SEM results in terms of cell adhesion and spreading at 1, 3, and 7 days after seeding. The number of viable cells increased over the analyzed time period. In addition, it is worth noting how the cell morphology changed over time, varying from a geometry with few ramifications to a thread-like geometry characterized by an increased number of ramifications (Supplementary Figure S5), This suggested the establishment of a higher number of cell–cell and cell–material interactions over time. As expected, a significant decrease in the shape factor was also evaluated over time by additional studies of cell adhesion and spreading based on CLSM images. A decrease in the shape factor clearly indicates better adhesion and spreading. However, when compared to 300/55/45-PCL scaffolds, at day 3 and day 7 a significantly lower shape factor was measured in the case of BCP-containing 300/55/45-PCL structures, even though at day 1 no significant differences were found between the two types of cell-scaffold constructs (Supplementary Figure S6).

**FIGURE 6:**
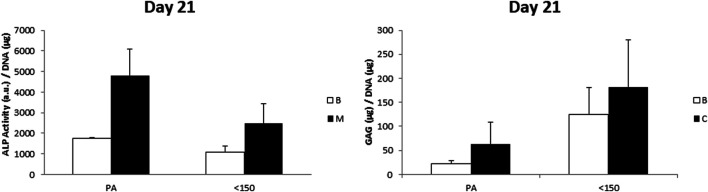
ALP/DNA and GAG/DNA measured for 300/55/45-PVP paste scaffolds sintered at a temperature lower than 150 °C, compared to smooth 300/55/45 scaffolds (PA). A general enhanced GAG production was observed on 300/55/45-PVP paste scaffolds, which was statistically significant (*p* < 0.05) in basic medium.

**FIGURE 7:**
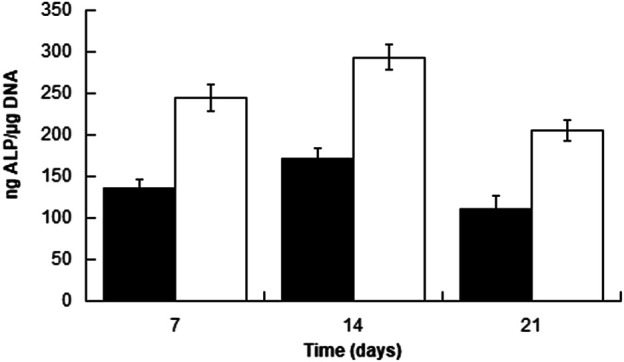
Normalized ALP activity (ALP/DNA) measured for 300PEOT55PBT45-PCL (black) and BCP-containing 300/55/45-PCL (white) scaffolds at different time points.

To further evaluate the possible influence of surface roughness on early hMSCs skeletal differentiation, we evaluated the ALP activity and GAG production at day 21 of 300/55/45-PVP pastes sintered lower than 150°C and compared to smooth scaffolds (Figure 6). Whereas there was no appreciable difference in case of ALP activity among the two scaffolds, a significant increase for GAG production was observed for 300/55/45-PVP pastes scaffolds. To further understand the potential of the developed scaffold library, we further investigated the ALP activity of 300/55/45-PCL pastes with or without BCP nanoparticles (Figure 7). For both groups of cell-scaffold constructs, a significant increase (*p* < 0.05) in the percentage of Alamar Blue reduction was evident from day 1 to day 7 (Supplementary Figure S4). As the magnitude of dye reduction may be generally related to the number of viable cells, the obtained results suggested that both kinds of scaffolds were able to support the adhesion and proliferation of hMSCs. Furthermore, no statistically significant differences (*p*>0.05) were found between 300/55/45-PCL and BCP-containing 300/55/45-PCL scaffolds at each time point. The normalized ALP activity (ALP/DNA) was determined at 7, 14 and 21 days in order to assess early osteogenic differentiation. The ALP/DNA ratio showed a peak value at 14 days for both 300/55/45-PCL and BCP-containing 300/55/45-PCL scaffolds. At each time point, higher values of normalized ALP activity were found for BCP-containing 300/55/45-PCL, compared to 300/55/45-PCL scaffolds. The observed differences were statistically significant (*p* < 0.05). These findings clearly demonstrated how the presence of BCP led to higher levels of ALP activity and provided an improvement in supporting the osteogenic differentiation of hMSCs.

Taken together, these results showed that surface modifications, in this particular case an increase in roughness, promoting cell proliferation, increased levels of metabolic activity, and attachment to the underlying material. Microsphere-based scaffolds are great injectable cell delivery systems that provide both large surface area to volume ratios, spatial and temporal control over drug release, and high packing degree (White et al., 2013; Rahman et al., 2014; Gupta et al., 2017). However, most constructs have limited functional groups on the surface of the constituent polymer to enable the grafting of biocompatible molecules. Gelatin methacrylate has recently been shown to be a biocompatible and photo cross-linkable macromolecule to be used as a modification of P_DL_LGA microspheres to enhance cell proliferation (Baki et al., 2017).

Wettability is another tailorable parameter regarding the construction of these structures. Hydrophilic materials often promote higher levels of cell attachment, with a spread and spindle-like shape; in contrast, more hydrophobic materials cause less cell attachment with rounded shape morphology (Mahmood et al., 2004). Control over cell density and morphology ultimately drives the quality of the regenerated tissue in a long term. Li et al. have improved the hydrophilic properties of PLGA-based microspheres using cross-linked gelatin to generate open pore microstructures to promote significant increases in cell metabolic activity (Li et al., 2015).

In addition to surface modifications, microspheres can be themselves modified and loaded with several drugs whose release can be tailored, including ibuprofen loaded PCL-microspheres (Carreras et al., 2013), ceftazidime loaded ethyl cellulose microspheres (Liu et al., 2010) and tetracycline loaded polyhydroxybutyrate microspheres (Meng et al., 2013) for bone regeneration, amongst other examples. In addition to the modifications on the microspheres *per se*, active biological components can also be incorporated in the polymeric binders if these are sintered at moderate temperatures that do not affect the bioactivity of those components. PCL has been one of the most been the most tailored polymer for this purpose. Chung et al. have functionalized PCL scaffolds by grafting nerve growth factor and Asp-Arg-Gly-Asp (GRGD) domains to promote growth and differentiation of neuronal cells (Chung et al., 2014). In the same line of studies, Rameshbabu et al. have embedded PCL-based nanofibers with placental-derived bioactive molecules to promote adhesion, infiltration, and proliferation of human keratocytes (Rameshbabu et al., 2018). Additionally, blends of two or more polymers can also be utilized (Washburn et al., 2002; Sarazin et al., 2004; Yuan and Favis, 2004). These systems would be particularly useful when complex engineering tissues are demanded with different cell types arranged in hierarchical structures, such as the heart, the liver and the neural tissue (Risbud et al., 2002; Cao et al., 2003). This approach would also be particularly suitable to engineer tissues on which it is essential to maintain the original cell morphology, such as for chondrocytes in cartilage tissue engineering (Mahmood et al., 2004) (Freed et al., 1993; Barry et al., 2004).

Physical modifications can also be introduced on microsphere-based scaffolds to potentiate their applicability on tissue engineering approaches. Zhou et al. have used co-electrospraying techniques to fabricate hollow PCL microspheres with and without a single surface hole (Zhou et al., 2017). The fabricated porous microspheres may allow cells to infiltrate to their interior as well as to the interstitial space among the microspheres, therefore facilitating cell–cell interactions within and between the microspheres.

Future studies should aim at further understanding the degradation profile of these pastes. Despite we have used biomaterials with well-known degradation kinetics, such as PVP, PCL, 300PEOT55PBT45, alginate, and BCP, it may well be that their combination in the presented paste formulation alters their degradation profile. It is frequently reported how the degradation kinetics of 3D scaffolds plays a crucial role in tissue engineering, as such structures should act as a support, also providing a suitable environment for the cells to grow inside and degrading at an appropriate rate, during and after the regeneration process. In literature, the potential of integrating several functionalities has also been explored for 3D additive manufactured scaffolds using several functionalization/bioactivation strategies. As earlier discussed, biomolecules and drugs may be directly immobilized on the scaffold fiber surface or loaded in the 3D interconnected pore network, as well as inside the microspheres. In this case, the drug release rate should be in accordance with the degradation rate.

Despite we have shown here that it is possible to tailor to a certain extent the surface roughness of polymeric paste scaffolds by varying the size of the microspheres, the type of polymeric carrier of the microspheres, the ratio between the microspheres and the polymeric carrier, and in some cases the sintering temperature and time used to bond the microspheres, future studies should also aim at further controlling the surface roughness of the resulting scaffolds. This may also provide interesting outcomes not only to steer cell activity, but also to prevent bacterial adhesion, which is known to be strongly dependent from surface roughness and topography of biomedical implants.

## Conclusion

We successfully created a family of polymeric pastes by mixing PEOT/PBT microspheres with different polymeric biocompatible binders, namely PVP, PCL, and alginate. 3DF scaffolds with a controlled pore network and surface roughness were fabricated. These structures can find diverse applications in tissue engineering and as drug delivery systems either by drug encapsulation or by surface adsorption.

Although microsphere-based techniques demonstrated to considerably improve the quality of tissue engineered constructs over the traditional additive manufacturing methods for creating microporous structures (Gupta et al., 2017, (Hutmacher et al., 2003; Schantz et al., 2005; Woodfield et al., 2005; Gupta et al., 2017), the high temperatures associated with the fabrication of the molten polymers still remain a critical drawback. The high temperatures required for sintering may compromise the biocompatibility of the biological factors to be incorporated within the scaffold structure. To overcome this, we used polymeric pastes that were processed and extruded at room temperature and several second phase polymers with low sintering temperatures.

## Data Availability

The raw data supporting the conclusions of this article will be made available by the authors upon reasonable requests, without undue reservation.
