# Supplementary material for: PEOT/PBT Polymeric Pastes to Fabricate Additive Manufactured Scaffolds for Tissue Engineering
Source: Front Bioeng Biotechnol. 2021 Sep 14;9:704185. doi: 10.3389/fbioe.2021.704185 (PMC8476768; doi:10.3389/fbioe.2021.704185)
Supplement: Supplementary file 1 [file DataSheet1.pdf]

Supplementary Information

PEOT/PBT polymeric pastes to fabricate additive manufactured scaffolds for tissue engineering

Gustavo A. Higuera [a], Tiago Ramos[b], Antonio Gloria[c], Luigi Ambrosio[c], Andrea Di Luca[a], Nicholas Pechkov[a], Joost R. de Wijn[a], Clemens A. van Blitterswijk[d], Lorenzo Moroni[d]

[a] Institute for BioMedical Technology and Technical Medicine (MIRA), Tissue Regeneration Department, University of Twente, P.O. Box 217, 7500 AE Enschede, The Netherlands.

[b] Institute of Ophthalmology, University College of London, 11-43 Bath Street, EC1V 9EL London, United Kingdom

[c] Institute of Polymers, Composites and Biomaterials, National Research Council of Italy, V.le J.F. Kennedy 54 - Pad. 20 Mostra d'Oltremare, 80125, Naples, Italy.

[d] MERLN Institute for Technology-inspired Regenerative Medicine, Complex Tissue Regeneration Department, Maastricht University, P.O. Box 616, 6200MD Maastricht, The Netherlands

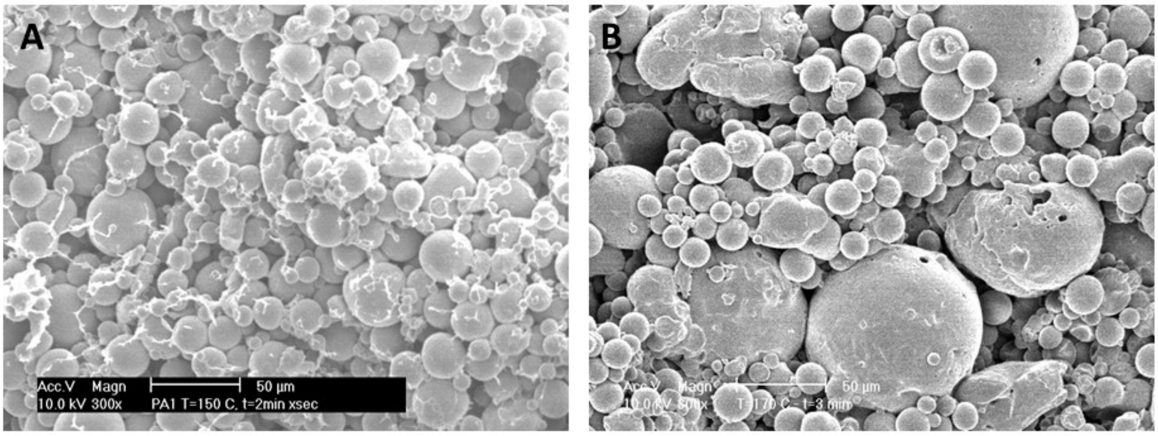

Figure S1. (A) 300PEOT55PBT45-PVP paste composed of microspheres with dimensions inferior to 64 µm. (B) 300PEOT55PBT45-PVP paste composed of microspheres with dimensions ranging from 64 to 128 µm. Scale bars: 50 µm.

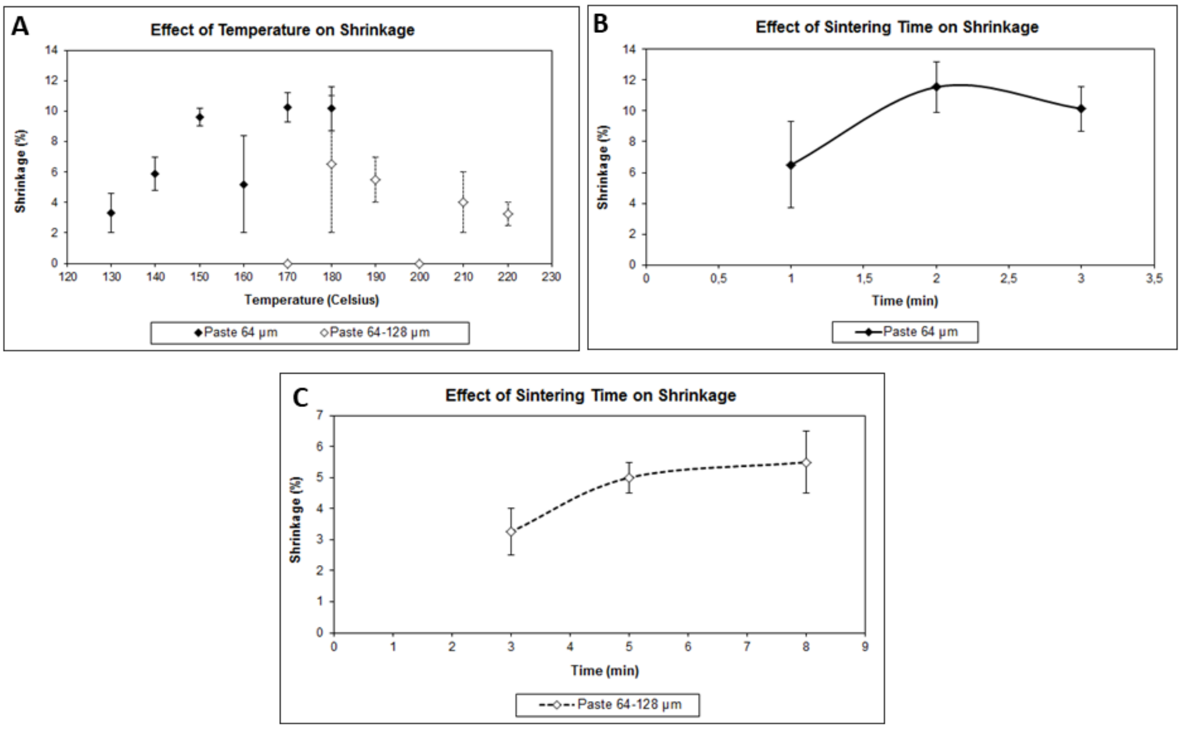

Figure S2. (A) Shrinkage of 300PEOT55PBT45-PVP pastes at different temperatures spanning from 130 to 220 C. Shrinkage of 300PEOT55PBT45-PVP pastes composed of microspheres with (A) dimensions below 64 um and (B) dimentions between 64 um and 128 um at different sintering times when the temperature was set to 180 C.

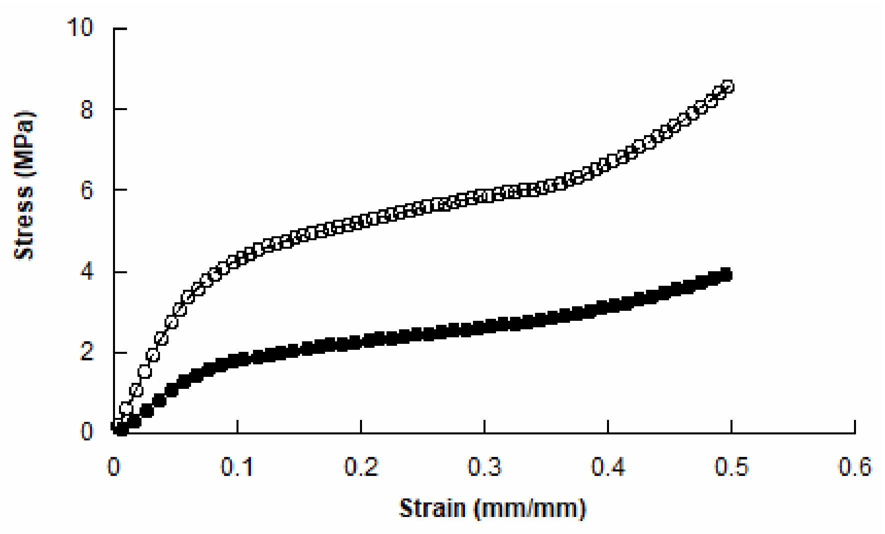

Figure S3. Compressive mechanical response of 3DF scaffolds based on PEOT/PBT polymeric pastes: typical stress-strain curves obtained for structures fabricated with 300PEOT55PBT45-PCL (black) and BCP-containing 300PEOT55PBT45-PCL (white) pastes (rate of 1 mm/min, final strain of 50%).

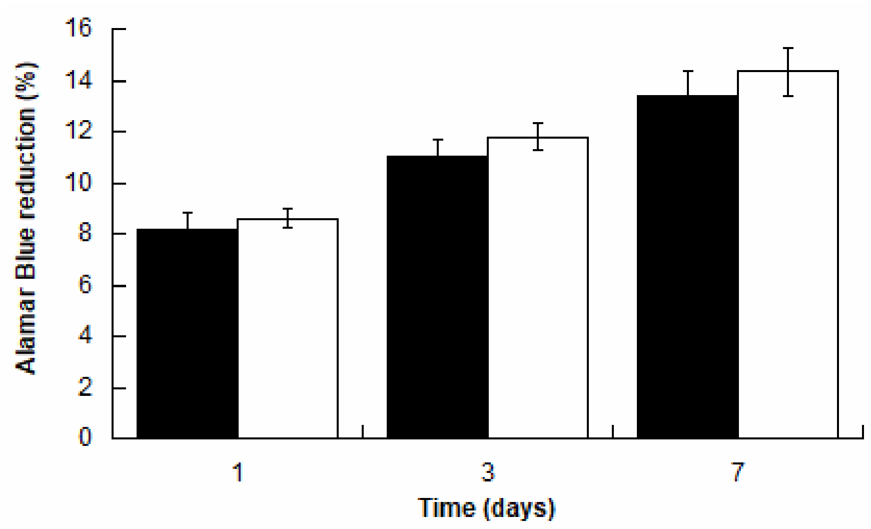

Figure S4. Alamar Blue reduction measured for 300PEOT55PBT45-PCL (black) and BCP-containing 300PEOT55PBT45-PCL (white) scaffolds at different time points.

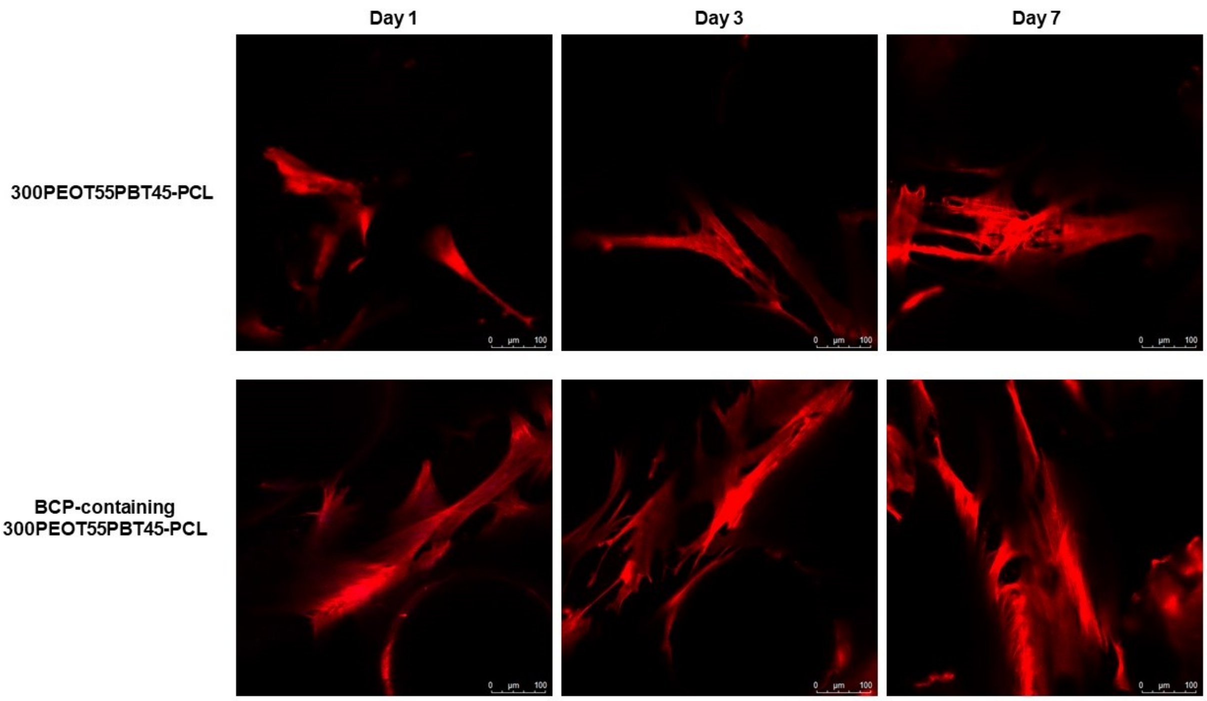

Figure S5. CLSM analysis on cell-scaffold constructs at different time points. Representative images of rhodamine phalloidin-stained actin filaments (red). Scale bar: 100 μm.

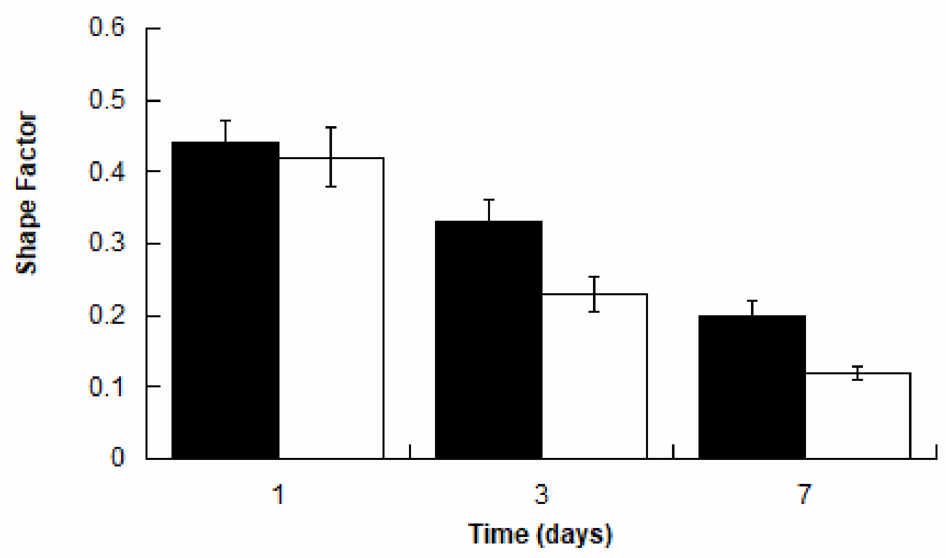

Figure S6. Typical values of shape factor obtained for hMSCs on 300PEOT55PBT45-PCL (black) and BCP-containing 300PEOT55PBT45-PCL (white) scaffolds at different time points.
